# Lateralization of epilepsy using intra‐hemispheric brain networks based on resting‐state MEG data

**DOI:** 10.1002/hbm.24990

**Published:** 2020-05-13

**Authors:** Haatef Pourmotabbed, James W. Wheless, Abbas Babajani‐Feremi

**Affiliations:** ^1^ Department of Orthopaedic Surgery and Biomedical Engineering University of Tennessee Health Science Center Memphis Tennessee USA; ^2^ Department of Biomedical Engineering University of Memphis Memphis Tennessee USA; ^3^ Department of Pediatrics, Division of Pediatric Neurology University of Tennessee Health Science Center Memphis Tennessee USA; ^4^ Neuroscience Institute & Le Bonheur Comprehensive Epilepsy Program Le Bonheur Children's Hospital Memphis Tennessee USA; ^5^ Department of Anatomy and Neurobiology University of Tennessee Health Science Center Memphis Tennessee USA

**Keywords:** focal epilepsy, functional connectivity, graph measures, intra‐hemispheric brain networks, machine learning, magnetoencephalography, network‐based statistics

## Abstract

Focal epilepsy originates within networks in one hemisphere. However, previous studies have investigated network topologies for the entire brain. In this study, magnetoencephalography (MEG) was used to investigate functional intra‐hemispheric networks of healthy controls (HCs) and patients with left‐ or right‐hemispheric temporal lobe or temporal plus extra‐temporal lobe epilepsy. 22 HCs, 25 left patients (LPs), and 16 right patients (RPs) were enrolled. The debiased weighted phase lag index was used to calculate functional connectivity between 246 brain regions in six frequency bands. Global efficiency, characteristic path length, and transitivity were computed for left and right intra‐hemispheric networks. The right global graph measures (GGMs) in the theta band were significantly different (*p* < .005) between RPs and both LPs and HCs. Right and left GGMs in higher frequency bands were significantly different (*p* < .05) between HCs and the patients. Right GGMs were used as input features of a Naïve‐Bayes classifier to classify LPs and RPs (78.0% accuracy) and all three groups (75.5% accuracy). The complete theta band brain networks were compared between LPs and RPs with network‐based statistics (NBS) and with the clustering coefficient (CC), nodal efficiency (NE), betweenness centrality (BC), and eigenvector centrality (EVC). NBS identified a subnetwork primarily composed of right intra‐hemispheric connections. Significantly different (*p* < .05) nodes were primarily in the right hemisphere for the CC and NE and primarily in the left hemisphere for the BC and EVC. These results indicate that intra‐hemispheric MEG networks may be incorporated in the diagnosis and lateralization of focal epilepsy.

## 1 INTRODUCTION

Epilepsy is a chronic brain disorder characterized by recurrent, transient interruptions of normal brain function in the form of hypersynchronous neuronal activity (Fisher et al., 2005). These interruptions, called seizures, can be classified as focal onset seizures (previously known as partial seizures), or generalized onset seizures (Berg et al., 2010). Focal onset seizures originate within networks limited to one cerebral hemisphere whereas generalized onset seizures originate within and rapidly engage bilaterally distributed networks (Berg et al., 2010). Because epilepsy is a disorder characterized by abnormal brain networks (Engel Jr. et al., 2013), various studies over the past decade have used both global and local graph measures to characterize these abnormal networks in patients with epilepsy. Brain networks are often constructed using functional connectivity (FC) or effective connectivity analysis based on interictal or ictal data from functional magnetic resonance imaging (fMRI), electroencephalography (EEG), magnetoencephalography (MEG), and intracranial EEG (iEEG).

Global graph measures characterize the integration, segregation, and integrity of networks as a whole whereas local graph measures characterize the properties of individual nodes and their influence on the network (Rubinov & Sporns, 2010). Global graph measures have been used to compare the functional networks of healthy controls (HCs) and patients with epilepsy with iEEG (Vega‐Zelaya, Pastor, de Sola, & Ortega, 2014), EEG (Douw et al., 2010; Horstmann et al., 2010; Quraan, McCormick, Cohn, Valiante, & McAndrews, 2013; van Diessen, Otte, Stam, Braun, & Jansen, 2016), fMRI (Doucet et al., 2015; Garcia‐Ramos, Song, Hermann, & Prabhakaran, 2016), and MEG (Chavez, Valencia, Navarro, Latora, & Martinerie, 2010; Horstmann et al., 2010; Niso et al., 2015). Global graph measures have also been used with MEG data to compare the functional networks of patients with epilepsy before and after resection surgery (van Dellen et al., 2014). Many studies have investigated a potential application of local graph measures, particularly those that denote the hub status of a node such as betweenness centrality (BC), in localizing the seizure onset and epileptogenic zones of patients with focal epilepsy to improve outcomes after epilepsy surgery. These localization studies have been performed using effective connectivity analysis with ictal and interictal iEEG (Ren et al., 2019; Wilke, Worrell, & He, 2011) and FC analysis with interictal EEG (Coito et al., 2019) and MEG (Juarez‐Martinez et al., 2018; Nissen et al., 2017; Nissen et al., 2018).

The aforementioned studies investigating the brain networks of patients with epilepsy have used global graph measures to characterize the network of the entire brain. Few studies have used global graph measures to investigate subnetworks that include only parts of the brain. These studies include using fMRI FC analysis to compare the inter‐ and intra‐hemispheric brain networks of patients with epilepsy before and after total callosotomy (Hung et al., 2019), using fMRI FC analysis to compare the default mode network of HCs and patients with temporal lobe epilepsy (TLE) that were seizure‐free and not seizure‐free after resection surgery (Ofer et al., 2019), and using EEG FC analysis to compare the intra‐hemispheric frontotemporal networks of HCs and patients with right or left frontotemporal epilepsy (Vecchio et al., 2015). Because focal onset seizures originate within networks limited to one hemisphere (Berg et al., 2010), global graph measures based on intra‐hemispheric networks may be able to reveal information about the brain networks of patients with focal epilepsy that global graph measures based on the network of the entire brain may not be able to.

In the current study, global graph measures based on FC analysis with resting‐state MEG (rs‐MEG) data were used to compare the intra‐hemispheric brain networks of HCs, patients with focal epilepsy originating from the left hemisphere (LPs), and patients with focal epilepsy originating from the right hemisphere (RPs). Machine learning was used to evaluate how well these global graph measures could be used to classify the three groups. Our hypothesis was that HCs, LPs, and RPs would have different intra‐hemispheric brain network topologies and that this difference could be demonstrated using FC analysis with rs‐MEG data. Network‐based statistics (NBS) was used to identify other subnetworks that may be different between LPs and RPs while local graph measures were used to identify brain regions that may have different network properties for LPs and RPs.

## 2 METHODS

### 2.1 Subjects

Approximately 1,000 patients with epilepsy underwent MEG data collection between 2007 and 2018 as part of the epilepsy evaluation process during their inpatient stay in the Epilepsy Monitoring Unit (EMU) at Le Bonheur Children's Hospital. From these patients, 41 patients with focal epilepsy (25 LPs, 16 RPs) were retrospectively selected for inclusion in this study. The inclusion criteria were patients: (a) who were diagnosed with TLE or temporal plus extra‐temporal lobe epilepsy (TELE) originating from either the left or right hemisphere, (b) for whom both anatomical MRI and rs‐MEG data were available, and (c) whose MEG data were not contaminated with artifacts generated by vagus nerve stimulation implantation, sedation, orthodontic devices, ventriculoperitoneal (VP) shunts, and/or environmental noise. About 80% of the patients did not meet the inclusion criteria due to the diagnosis of generalized epilepsy, the occurrence of bilateral discharges, lack of anatomical MRI or rs‐MEG data, and/or the presence of artifacts. The diagnosis of focal epilepsy was based on clinical information, seizure semiology, potential lesions in anatomical MRI data, and localization of seizures or interictal epileptiform discharges (IEDs) from video‐EEG monitoring, MEG data, and/or single‐photon emission computerized tomography (SPECT) data. To promote homogeneity in the two patient groups and ensure correct lateralization of focal epilepsy, patients in the remaining 20% were included only if there was successful localization of epileptiform discharges to the left or right temporal lobe and the results were conclusive across all the modalities. The study was approved by the Institutional Review Board (IRB) of the University of Tennessee Health Science Center. Demographic and clinical data for the patients are shown in Table 1.

**Table 1:** Demographic and clinical data for the patients

	Left patients (*n* = 25)	Right patients (*n* = 16)	*p*‐values^a^
Age (mean ± *SD* years)	16.6 ± 6.2	16.9 ± 8.0	.894
Age at seizure onset (mean ± *SD* years)	8.7 ± 7.9	8.5 ± 5.7	.929
Gender (M, F)	13, 12	10, 6	.540
Focal region (TLE, TELE)	7, 18	6, 10	.732
MRI status (Normal, FCD, MTS, other)	3, 6, 3, 13	3, 4, 1, 8	.927
Language laterality (left, right, bilateral, unknown)	11, 7, 3, 4	11, 3, 0, 2	.369
Number of current AEDs (median ± IQR)^b^	2 ± 1	1.5 ± 1	.811

Abbreviations: FCD, focal cortical dysplasia; MTS, mesial temporal lobe sclerosis; TELE, temporal plus extra‐temporal lobe epilepsy; TLE, temporal lobe epilepsy.

a To test for a significant difference between the left and right patients, a two sample *t*‐test was used for the age and age at seizure onset, Fisher's exact test for gender and focal region, the Freeman–Halton extension of Fisher's exact test for MRI status and language laterality, and the Wilcoxon rank sum test for the number of anti‐epileptic drugs (AEDs).

b The AEDs include levetiracetam, ethosuximide, clonazepam, clobazam, rufinamide, lamotrigine, lacosamide, zonisamide, carbamazepine, oxcarbazepine, valproic acid, phenytoin, tiagabine, and topiramate.

A total of 22 HCs (25.8 ± 5.1 [mean ± *SD*] years; 10 females) who voluntarily participated for anatomical MRI and rs‐MEG data collection at Le Bonheur Children's Hospital between 2017 and 2018 were also included in this study. Most of the HCs were left hemisphere dominant for language (10 left, 6 right, 6 bilateral). The HCs were free from MRI‐detected lesions and any significant neurodevelopmental (e.g., autism), neuropsychiatric (e.g., depression), or neurologic (e.g., epilepsy) disorders.

### 2.2 Software note

The FieldTrip toolbox v20180905 (Oostenveld, Fries, Maris, & Schoffelen, 2011) was used to preprocess the MEG data, co‐register the MEG data with the anatomical MRI, perform beamformer virtual electrode (VE) reconstruction, and perform FC analysis. The Brain Connectivity Toolbox v20170115 (Rubinov & Sporns, 2010) and Network‐Based Statistic toolbox v1.2 (Zalesky, Fornito, & Bullmore, 2010) were used to perform network analysis. The BrainNet Viewer toolbox v20150123 (Xia, Wang, & He, 2013) was used to visualize the brain networks. The Statistics and Machine Learning Toolbox of MATLAB (MathWorks Inc., Natick, MA) was used to perform machine learning. All analyses were performed using MATLAB R2018b.

### 2.3 Data acquisition and preprocessing

A whole‐head 248‐magnetometer MAGNES 3600 MEG system (4D Neuroimaging, San Diego, CA) housed in a magnetically shielded room at Le Bonheur Children's Hospital was used to collect resting‐state MEG data from all subjects in a supine position (eyes‐open for the HCs and eyes‐closed for the patients). A 1017.25 Hz sampling rate (with no online filter) was used for all HCs, a 678.17 Hz sampling rate (with no online filter) was used for 27 patients, and a 508.63 Hz sampling rate (with an online 0.1 Hz highpass filter) was used for 14 patients. Five head position indicator (HPI) coils, three of which were anchored to three anatomical landmarks (i.e., the per‐auricular points and nasion) and two on the forehead, were used to determine the head's position during the data collection. The scalp outline (i.e., head shape) and HPI coil positions were digitized using a 3D digitizer (Fastrak, Polhemus, Colchester, VT). A 3T Siemens Verio scanner (Siemens AG, Munich, DE) or a 3T Signa HDxt scanner (General Electric Healthcare, Milwaukee, WI) at Le Bonheur Children's Hospital was used to obtain high‐resolution T1‐weighted anatomical MRI images, which were co‐registered with the MEG data using surface‐matching software. The anatomical landmarks and scalp outline were used to guide co‐registration of the MRI data with the MEG data. The co‐registered MRI was segmented, and the brain surface from the segmented MRI was used to compute a realistic single‐shell volume conductor model (Nolte, 2003) for calculation of the lead‐fields.

**Figure 1:**
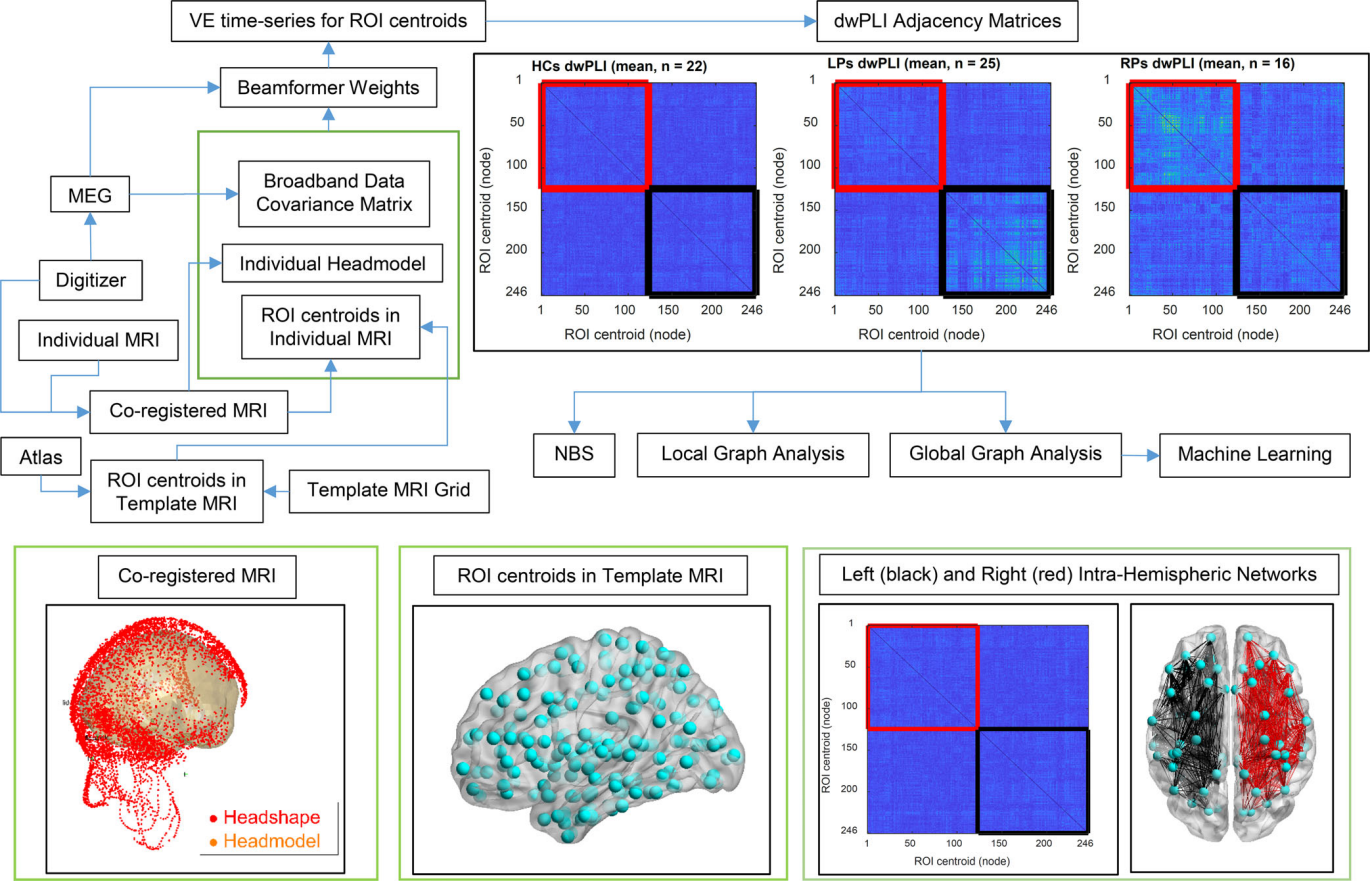
Overview of the analysis pipeline. Abbreviations: ROI, region of interest; VE, virtual electrode; dwPLI, debiased weighted phase lag index; HCs, healthy controls; LPs, patients with left‐hemispheric focal epilepsy; RPs, patients with right‐hemispheric focal epilepsy; NBS, network‐based statistics

The MEG data were 0.1–150 Hz bandpass filtered, 60 and 120 Hz bandstop filtered, and segmented into 3‐s trials. Trials and sensors containing artifacts and epileptiform activity were removed via visual inspection according to temporal variance, z‐score, and kurtosis outliers. Trials with epileptiform activity were removed according to conventional practice, as seen in previous studies (Chavez et al., 2010; Juarez‐Martinez et al., 2018; Niso et al., 2015; Nissen et al., 2017; van Diessen et al., 2016). Removed sensors were reconstructed via spherical spline interpolation (Perrin, Pernier, Bertrand, & Echallier, 1989). Due to technical problems, three channels were discarded from the analysis for some of the patients. For FC analysis in the delta band, consecutive 3‐s trials were placed together to form 12‐s trials. After artifact rejection, the MEG data of all the subjects contained from 73 to 190 3‐s trials (3.65 to 9.5 min) and from 16 to 46 12‐s trials (3.2 to 9.2 min). An overview of the analyses performed on the MEG data is shown in Figure 1.

### 2.4 Beamformer VE reconstruction for regions of interest

The pre‐processed MEG data were projected through beamformer weights to reconstruct VE time‐series for the centroids of the 246 (210 cortical, 36 subcortical) regions of interest (ROIs) defined on the Brainnetome atlas (Fan et al., 2016). The beamformer weights were computed for each centroid separately using the regularized broadband (0.1–150 Hz) data covariance matrix and the lead‐fields calculated from the volume conductor model. The weights form a spatial filter that allows signals to pass from the location of interest and attenuate signals from all other locations.

Voxels in a 2‐mm resolution template MRI grid in the Montreal Neurological Institute (MNI) space were labeled according to the Brainnetome atlas, and voxels with the same label were defined as a ROI. For each ROI, the *k*‐medoids algorithm was used to locate the voxel, designated as the centroid, with the minimal squared Euclidean distance to all other voxels of the ROI. These centroids were nonlinearly warped to the co‐registered MRI of each subject. For the construction of the beamformer weights, a time window that included all the trials that remained after artifact rejection was used to compute the broadband data covariance matrix, as per the suggestion made in Brookes et al. (2008). The covariance matrix was regularized using the Tikhonov method with a noise floor equal to 5% of the average of the eigenvalues of the matrix.

The beamformer output, called a VE, at a target source location and orientation θ is the weighted sum of the signals of all the MEG sensors (van Veen, van Drongelen, Yuchtman, & Suzuki, 1997):

$$V_{\theta }=B∙W_{\theta }$$

where V_θ_ is the N × 1 VE vector for all N time points, B is the N × M data matrix containing the magnetic field at the M sensor locations for all N time points, and W_θ_ is the M × 1 weight vector. The weight vector is computed according to the following formula (Mosher, Baillet, & Leahy, 2003; Robinson & Vrba, 1999; van Drongelen, Yuchtman, Van Veen, & van Huffelen, 1996; van Veen et al., 1997):

$$W_{\theta }=\frac{C_{b}^{-1}L_{\theta }}{L_{\theta }^{T}C_{b}^{-1}L_{\theta }}$$

where C_b_ is the M × M covariance matrix, L_θ_ is the M × 1 lead‐field vector, and T denotes matrix transpose. The optimum source orientation for the lead‐field vector was determined via eigendecomposition of the beamformer‐derived source covariance matrix, as is described in Sekihara, Nagarajan, Poeppel, and Marantz (2004). The beamformer‐derived source covariance matrix for location r is given in the following formula:

$$P\left(r\right)={\left[L^{T}\left(r\right)C_{b}L\left(r\right)\right]}^{-1}$$

where P(r) is the p × p source covariance matrix for p source orientation components and L(r) is the M × p lead‐field matrix.

### 2.5 FC analysis

Functional interactions between oscillating source activities can be obtained by quantifying the phase relationship between their time‐series. The phase lag index (PLI) measures the asymmetry in the distribution of instantaneous phase differences between two time‐series and is computed according to the following formula (Stam, Nolte, & Daffertshofer, 2007):

Ψf=EsgnImskf

where Ψ(f) is the PLI between two time series at frequency f, s_k_(f) is the cross‐spectrum between the time‐series for trial k at frequency f, Im[s_k_(f)] denotes the imaginary part of the cross‐spectrum, and E[.] is the expected value operator. The PLI is sensitive against the effects of noise sources, volume conduction, and field spread because it discards connections with a zero (modulus π)‐phase lag at the expense of potentially discarding true zero‐phase interactions (Stam et al., 2007). However, the sensitivity of the PLI is affected by the discontinuity in the index as low‐amplitude perturbations may change the sign of the phase difference between two time‐series for a particular trial (Vinck, Oostenveld, van Wingerden, Battaglia, & Pennartz, 2011). The weighted PLI (wPLI) has been proposed to reduce the effects of this discontinuity by weighting the sign of the phase difference with the magnitude of the imaginary part of the cross‐spectrum (Vinck et al., 2011):

Φf=EImskfEImskf

where Φ(f) is the wPLI between two time series at frequency f. As both the PLI and wPLI are affected by the number of trials used to compute them, the debiased wPLI‐square estimator (dwPLI) has been proposed to reduce the effects of this sample size bias and is given in the following formula (Vinck et al., 2011):

Ω^wf=∑k=1K∑j≠kImskf∙Imsjf∑k=1K∑j≠kImskf∙Imsjf

where ${\hat{\Omega }}^{w}\left(f\right)$ is the dwPLI between two time‐series at frequency f and K is the number of trials.

In the current study, the dwPLI was used to estimate FC between the ROI centroids. To compute the dwPLI between each pair of ROI centroids, Fourier spectra for each trial of the VE time‐series was obtained using the discrete Fast Fourier Transform with a Hann window and used to calculate the cross‐spectra between each pair of ROI centroids. 3‐s trials were used for frequencies above 3 Hz, and 12‐s trials were used for frequencies from 0.5 to 3 Hz. The dwPLI were averaged over the delta (0.5–3 Hz), theta (4–7 Hz), alpha (8–13 Hz), low beta (13–20 Hz), high beta (20–30 Hz), and low gamma (30–50 Hz) frequency bands and used to construct 246‐by‐246 weighted adjacency matrices.

### 2.6 Global graph analysis and machine learning

To characterize the difference between the intra‐hemispheric brain networks of the three groups, the second and fourth quadrants (123‐by‐123) of the adjacency matrices, which respectively represent the right and left intra‐hemispheric connection strengths between the ROI centroids (i.e., nodes), were set to a 10% threshold and used to compute three global graph measures (i.e, global efficiency (GE), characteristic path length (CPL), and transitivity (T)) in all six frequency bands. The length between two nodes was computed as the inverse of their connection strength, as per the suggestion made in Boccaletti, Latora, Moreno, Chavez, and Hwang (2006), and Dijkstra's algorithm was used to find the shortest path length. For nodes that were disconnected from each other, the shortest path length was set to infinity and excluded from the computation of the CPL. The CPL and GE are measures of functional integration where the CPL is defined as the average shortest path length between all pairs of nodes in the network (Watts & Strogatz, 1998) and the GE as the average inverse shortest path length (Latora & Marchiori, 2001). The GE is mainly influenced by short paths, which imply a stronger potential for functional integration between brain regions, while the CPL is mainly influenced by long paths (Rubinov & Sporns, 2010). T is a measure of functional segregation that represents the prevalence of clustered connectivity around the nodes in the network and is defined as the normalized sum of the weighted geometric mean of the connection strengths of all the triangles around all the nodes (Newman, 2003; Onnela, Saramaki, Kertesz, & Kaski, 2005; Rubinov & Sporns, 2010). T is a variant of the mean clustering coefficient (CC) that is normalized collectively and therefore does not suffer from the problem where the mean CC may be disproportionately influenced by nodes that have a few neighbors (Newman, 2003; Rubinov & Sporns, 2010). The Wilcoxon rank sum test was used to test for a significant difference (*p* < .05) between the global graph measures of the three groups. *p*‐values were false discovery rate (FDR) adjusted (Benjamini & Hochberg, 1995) for six frequency bands, three global graph measures, and two quadrants.

The global graph measures that were significantly different (*p* < .005) between LPs and RPs (i.e., the GE, CPL, and T in the theta band of the right intra‐hemispheric network) were used as input features of a Naïve‐Bayes classifier to classify the LPs and RPs. The global graph measures of the right intra‐hemispheric network that were significantly different (*p* < .01) between at least two of the three groups (i.e., the GE in the theta, low beta, and high beta bands [three features] and the CPL and T in the theta, low beta, high beta, and low gamma bands [8 features]) were used as input features of a Naïve‐Bayes classifier to classify the three groups.

To train and test the classifiers, the following procedure was used. The subjects were randomly partitioned and separated into 10‐folds. For each of 10 iterations, subjects in one fold were used as a testing‐set and subjects in the other nine folds were used as a training set. This procedure was repeated 100 times. The performance of the classifiers was evaluated by averaging the accuracy, sensitivity, specificity, precision, and area under the receiver operating characteristic (ROC) curve (AUC) over the 10 iterations and the 100 random partitions.

Although there was no significant difference between the hemispheric language dominance of the three groups (*p* > .05), language dominance may still influence the topology of the intra‐hemispheric networks. Therefore, an unbalanced two‐way analysis of variance (ANOVA) was performed to test whether the effect of language dominance on the global graph measures was significant. *p*‐values were FDR adjusted (Benjamini & Hochberg, 1995) for six frequency bands, three global graph measures, and two quadrants.

### 2.7 Local graph analysis and NBS

To characterize the difference between the brain networks of LPs and RPs on a local level, the complete adjacency matrices were set to a 10% threshold and used to compute four local graph measures (i.e., BC, eigenvector centrality [EVC], CC, and nodal efficiency [NE]) in the theta band. BC is based on the idea that central nodes (i.e., hubs) act as important controllers of information flow by participating in many short paths and is defined as the fraction of all the shortest paths in the network that pass through the node of interest (Freeman, 1979; Rubinov & Sporns, 2010). EVC is based on the idea that a node is more influential if it is connected to a few highly connected nodes rather than if it is connected to many poorly connected nodes and is defined as the element of the eigenvector of the largest eigenvalue of the adjacency matrix that corresponds to the node (Bonacich, 1972, 2007). Nodes have a high EVC if they are connected to other nodes that have a high EVC (Bonacich, 1972). The CC, a local variant of the T, is a measure of the amount of clustered connectivity around a node and is defined as the weighted geometric mean of the connection strengths of all the triangles around the node normalized by the total number of possible connections between the neighbors of the node (Onnela et al., 2005; Rubinov & Sporns, 2010; Watts & Strogatz, 1998). The NE, a local variant of the GE, is a measure of the ability of a node to send information to other nodes in the network and is defined as the average inverse shortest path length between the node and all other nodes (Latora & Marchiori, 2001; Rubinov & Sporns, 2010). The Wilcoxon rank sum test was used to test for a significant difference (*p* < .05) between the local graph measures of the LPs and RPs. *p*‐values were FDR adjusted (Benjamini & Hochberg, 1995) for one frequency band (i.e., the theta band), four local graph measures, and 246 nodes.

To identify a subnetwork that differs between LPs and RPs, NBS with a 3.45 primary threshold, 5,000 random permutations, and a *p* < .05 significance level (family‐wise error rate [FWER]‐corrected) (Zalesky et al., 2010) was performed on the connection strengths of the complete, un‐thresholded adjacency matrices of the LPs and RPs in the theta band. First, a mass univariate *t‐*test is performed on the connection strengths, resulting in a *t*‐statistic per connection. Only those connections with a *t*‐statistic greater than the primary threshold are admitted to the next step. Next, topological clusters are identified from the supra‐threshold connections, and permutation testing is used to compute FWER‐corrected *p*‐values for the identified clusters. During permutation testing, 5,000 random permutations are performed where the adjacency matrices of the patients are shuffled between the two groups. For each permutation, the process of performing a mass univariate *t*‐test, choosing supra‐threshold connections, and identifying topological clusters from the supra‐threshold connections is repeated. The size of the largest identified cluster for each permutation is used to generate an empirical null distribution. Therefore, the FWER‐corrected *p*‐value for a cluster of a given size represents the probability of randomly finding a cluster with the same or greater size under the null hypothesis.

To examine the similarity between the local graph measures and the subnetwork identified using NBS, the ROIs were grouped into 48 brain areas (24 in each hemisphere) according to the grouping scheme of the Brainnetome atlas, and the number of nodes in each brain area that had significant local graph measures or that were part of the NBS subnetwork was recorded.

## 3 RESULTS

### 3.1 Global graph analysis and machine learning

**Figure 2:**
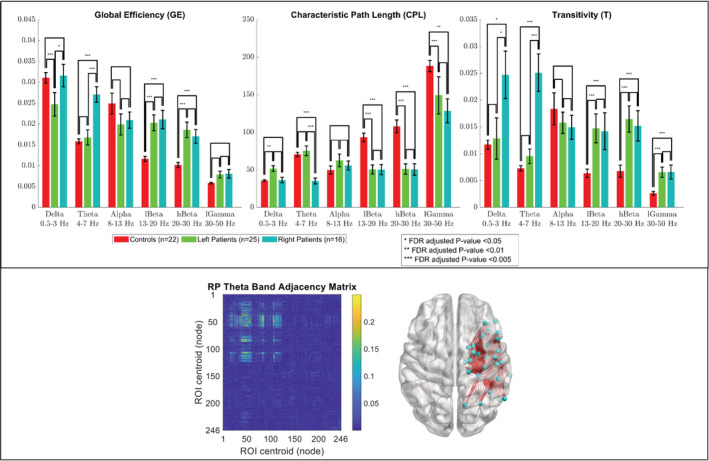
Group comparison of the global efficiency (GE), characteristic path length (CPL), and transitivity (T) of the second quadrant (i.e., right intra‐hemispheric network) of the adjacency matrix in six frequency bands. Bar lengths indicate mean values; error bars indicate standard errors of the mean. *p*‐values were false discovery rate (FDR) adjusted for six frequency bands, three global graph measures, and two quadrants. (Bottom insert) Adjacency matrix of a representative right patient in the theta band, and a corresponding anatomical depiction showing connections with values above 0.1

**Figure 3:**
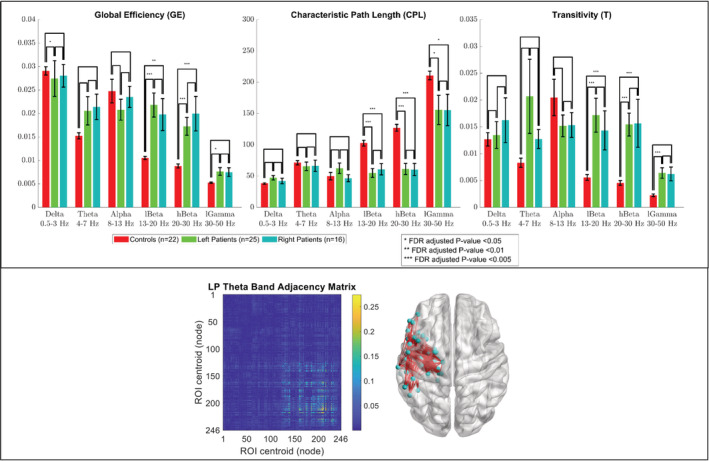
Group comparison of the global efficiency (GE), characteristic path length (CPL), and transitivity (T) of the fourth quadrant (i.e., left intra‐hemispheric network) of the adjacency matrix in six frequency bands. Bar lengths indicate mean values; error bars indicate standard errors of the mean. *p*‐values were false discovery rate (FDR) adjusted for six frequency bands, three global graph measures, and two quadrants. (Bottom insert) Adjacency matrix of a representative left patient in the theta band, and a corresponding anatomical depiction showing connections with values above 0.1

A group comparison of the GE, CPL, and T of the right and left intra‐hemispheric networks in all frequency bands for the LPs, RPs, and HCs is shown in Figure 2 and Figure 3. The results showed that the GE, CPL, and T of the right intra‐hemispheric network in the theta band were significantly different (*p* < .005, FDR‐adjusted) between the RPs and both the LPs and HCs with the GE and T being significantly greater for the RPs and the CPL being significantly lower. The GE of the right intra‐hemispheric network in the delta band was significantly lower (*p* < .05, FDR‐adjusted) for the LPs than the RPs and HCs while the T of the right intra‐hemispheric network in the delta band was significantly greater (*p* < .05, FDR‐adjusted) for the RPs than the LPs and HCs. For the LPs and RPs, there was no significant difference between the GE, CPL, and T of the right intra‐hemispheric network in the higher frequency bands (i.e., the alpha, low beta, high beta, and low gamma bands) and of the left intra‐hemispheric network in all frequency bands. The GE, CPL, and T of both intra‐hemispheric networks in the low and high beta bands were significantly different (*p* < .01, FDR‐adjusted) between the HCs and both patient groups with the GE and T being significantly lower for the HCs and the CPL being significantly greater. The CPL in both intra‐hemispheric networks in the low gamma band was significantly greater (*p* < .05, FDR‐adjusted) for the HCs than both patient groups while the T of the right intra‐hemispheric network in the low gamma band was significantly lower (*p* < .005, FDR‐adjusted) for the HCs than both patient groups. For the three groups, there was no significant difference between the GE, CPL, and T of both intra‐hemispheric networks in the alpha band. An unbalanced two‐way ANOVA showed that the effect of hemispheric language dominance on the global graph measures was not significant for any of the frequency bands.

When used as input features for a Naïve‐Bayes classifier to classify the LPs and RPs, the global graph measures that were significantly different (*p* < .005, FDR‐adjusted) between LPs and RPs (i.e., the GE, CPL, and T of the right intra‐hemispheric network in the theta band) were able to achieve a 78.0 ± 0.5% (mean ± *SD*) accuracy and an AUC of 0.869 ± 0.007 for both patient groups. When used as input features for a Naïve‐Bayes classifier to classify the three groups, the global graph measures of the right intra‐hemispheric network that were significantly different (*p* < .01, FDR‐adjusted) between at least two of the three groups (i.e., the GE in the theta, low beta, and high beta bands [three features] and the CPL and T in the theta, low beta, high beta, and low gamma bands [eight features]) were able to achieve a 75.5 ± 1.7% accuracy and an AUC of 0.988 ± 0.003 for the HCs, 0.837 ± 0.011 for the LPs, and 0.854 ± 0.009 for the RPs. The sensitivity, specificity, precision, and AUC, which were used to evaluate the performance of the classifiers, are shown in Table 2 and Table 3.

**Table 2:** Results of a Naïve‐Bayes classifier (78.0 ± 0.5% [mean ± *SD*] accuracy) in classifying patients with right‐hemispheric focal epilepsy (RPs) and patients with left‐hemispheric focal epilepsy (LPs) using the following three input features: the global efficiency (GE), characteristic path length (CPL), and transitivity (T) in the theta band of the right intra‐hemispheric network

	Sensitivity	Specificity	Precision	AUC^a^
Left patients (LRs)	0.760 ± 2.2E−16	0.811 ± 0.013	0.863 ± 0.008	0.869 ± 0.007
Right patients (RPs)	0.811 ± 0.013	0.760 ± 2.2E−16	0.684 ± 0.003	0.869 ± 0.007
Weighted average	0.780 ± 0.005	0.791 ± 0.008	0.793 ± 0.006	0.869 ± 0.007

a AUC = area under the receiver operating characteristic (ROC) curve.

**Table 3:** Results of a Naïve‐Bayes classifier (75.5 ± 1.7% [mean ± *SD*] accuracy) in classifying healthy controls (HCs), patients with right‐hemispheric focal epilepsy (RPs), and patients with left‐hemispheric focal epilepsy (LPs) using the following 11 input features: the global efficiency (GE) in the theta, low beta, and high beta bands; the characteristic path length (CPL) in the theta, low beta, high beta, and low gamma bands; and the transitivity (T) in the theta, low beta, high beta, and low gamma bands of the right intra‐hemispheric network

	Sensitivity	Specificity	Precision	AUC^a^
Healthy controls (HCs)	0.881 ± 0.038	0.955 ± 0.009	0.913 ± 0.016	0.988 ± 0.003
Left patients (LPs)	0.651 ± 0.024	0.823 ± 0.024	0.708 ± 0.029	0.837 ± 0.011
Right patients (RPs)	0.743 ± 0.024	0.854 ± 0.012	0.634 ± 0.021	0.854 ± 0.009
Weighted average	0.755 ± 0.017	0.877 ± 0.010	0.761 ± 0.015	0.894 ± 0.006

a AUC = area under the receiver operating characteristic (ROC) curve.

### 3.2 Local graph analysis and NBS

**Figure 4:**
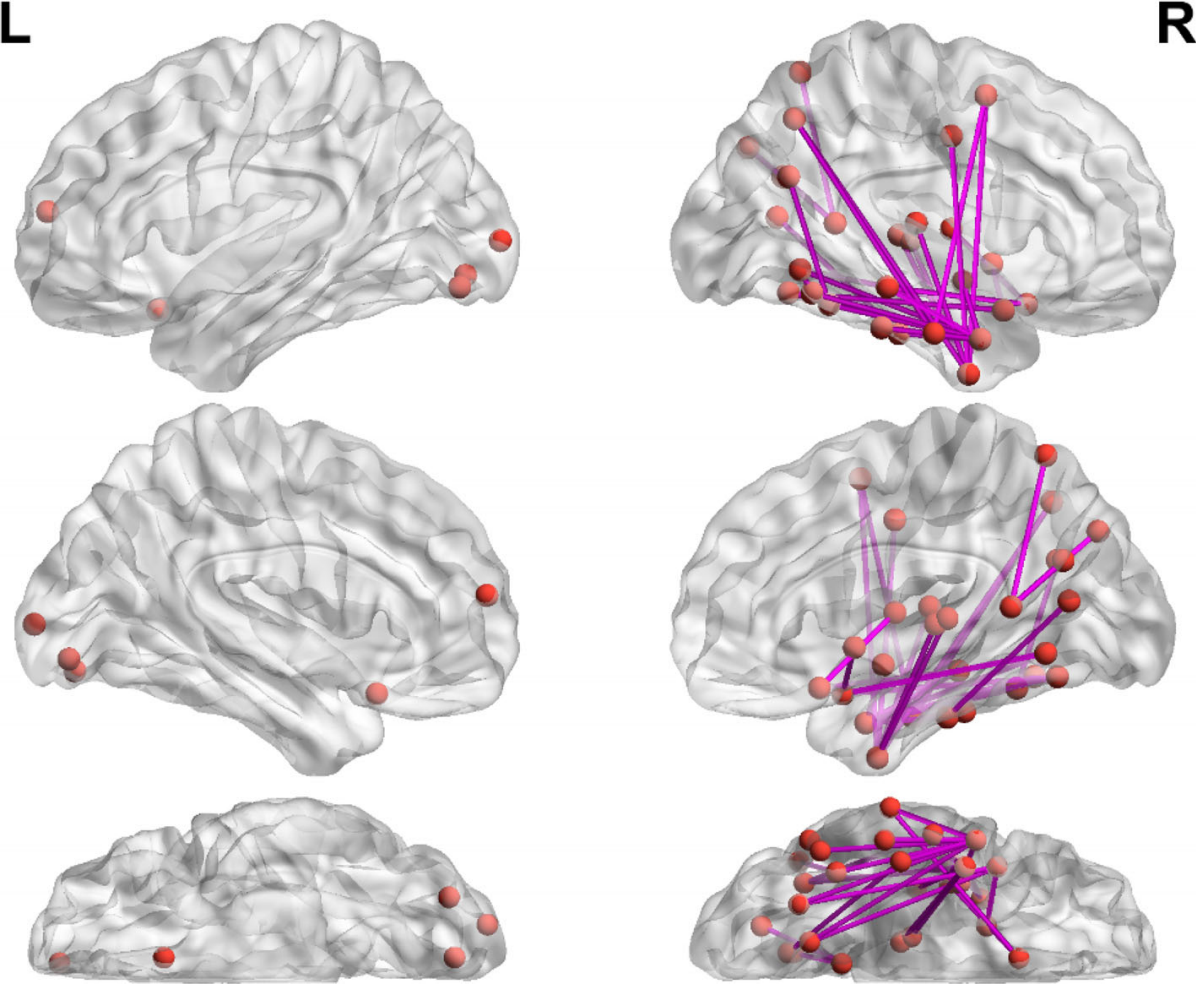
Subnetwork identified using network‐based statistics (NBS) with a 3.45 primary threshold, 5,000 random permutations, and a *p* < .05 significance level (family‐wise error rate [FWER]‐corrected) on the connection strengths of the complete, un‐thresholded adjacency matrices of patients with right‐hemispheric focal epilepsy (RPs) and patients with left‐hemispheric focal epilepsy (LPs) in the theta band. The network was primarily composed of intra‐hemispheric connections within the right hemisphere (27 were right intra‐hemispheric and 8 interhemispheric) and had 28 nodes located in the right hemisphere (3 were frontal, 11 temporal, 4 parietal, 3 insular, 1 limbic, 3 occipital, 1 basal ganglia, and 2 thalamus) and 5 nodes located in the left hemisphere (2 were frontal and 3 occipital). The most significant connections were intra‐hemispheric connections in the right hemisphere between two nodes in the inferior temporal gyrus (ITG) (*t* = 4.47), a node in the ITG and a node in the inferior parietal lobule (IPL) (*t* = 4.20), a node in the fusiform gyrus (FG) and a node in the IPL (*t* = 4.18), a node in the superior temporal gyrus (STG) and a node in the basal ganglia (BG) (*t* = 4.09), and a node in the ITG and a node in the IPL (*t* = 4.00)

Performing NBS with a 3.45 primary threshold, 5,000 random permutations, and a *p* < .05 significance level (FWER‐corrected) on the connection strengths of the complete, un‐thresholded adjacency matrices of RPs and LPs in the theta band was able to identify a subnetwork, which is shown in Figure 4, that differs between the two patient groups. The network was primarily composed of intra‐hemispheric connections within the right hemisphere (27 were right intra‐hemispheric and 8 interhemispheric) and had 28 nodes located in the right hemisphere (11 were temporal and 3 subcortical), and 5 nodes located in the left hemisphere (2 were frontal and 3 occipital). The most significant connections were intra‐hemispheric connections in the right hemisphere between two nodes in the inferior temporal gyrus (ITG) (*t* = 4.47), a node in the ITG and a node in the inferior parietal lobule (IPL) (*t* = 4.20), a node in the fusiform gyrus (FG) and a node in the IPL (*t* = 4.18), a node in the superior temporal gyrus (STG) and a node in the basal ganglia (BG) (*t* = 4.09), and a node in the ITG and a node in the IPL (*t* = 4.00).

**Figure 5:**
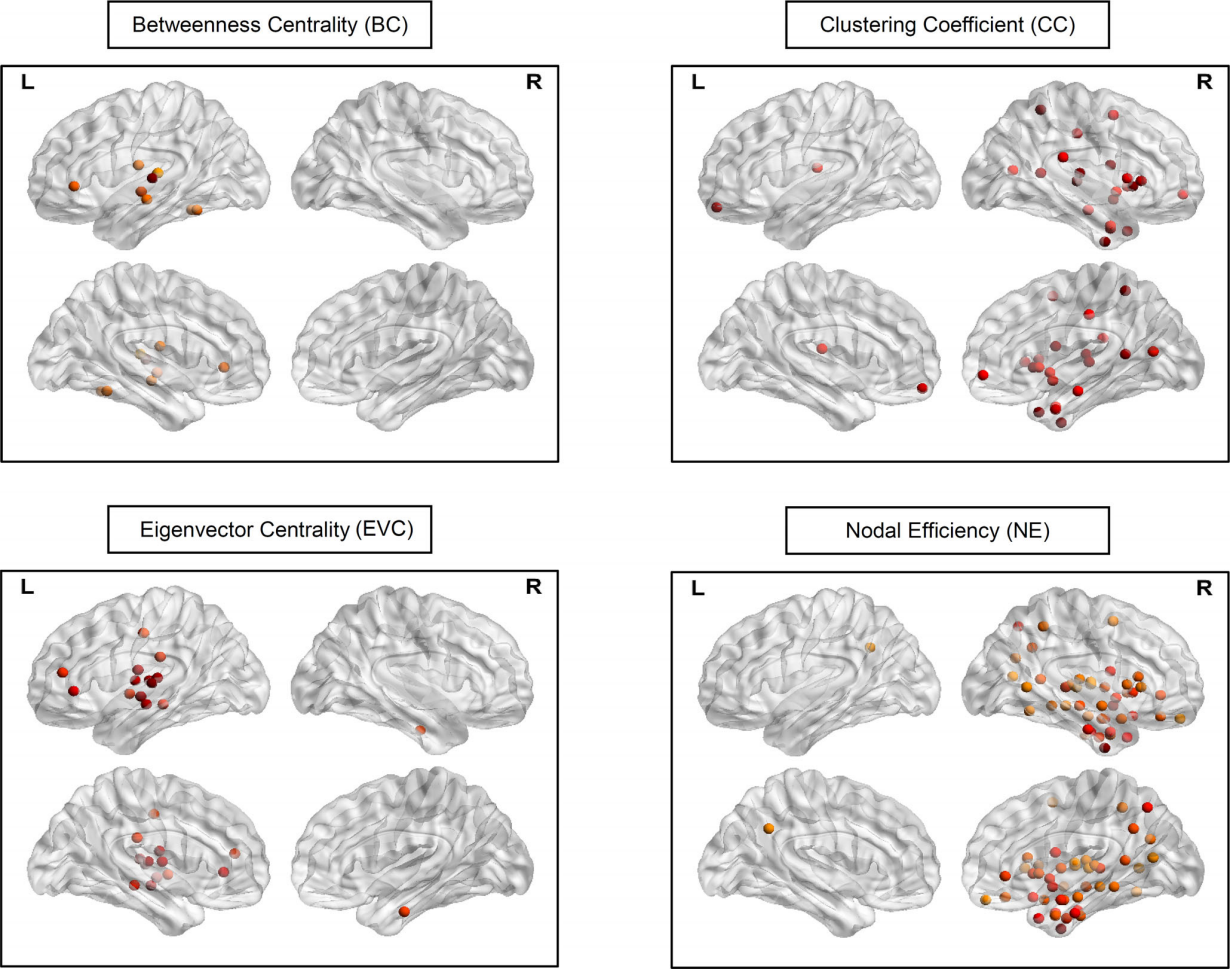
Significant nodes (*p* < .05) identified via comparison of the betweenness centrality (BC), eigenvector centrality (EVC), clustering coefficient (CC), and nodal efficiency (NE) of the complete adjacency matrices in the theta band of patients with right‐hemispheric focal epilepsy (RPs) and patients with left‐hemispheric focal epilepsy (LPs). *p*‐values were false discovery rate adjusted for one frequency band (i.e., the theta band), four local graph measures, and 246 nodes. The color of the nodes are scaled according to their *z*‐values, with a darker color indicating a greater value. For BC, there were 8 nodes located in the left hemisphere (1 was frontal, 6 temporal, and 1 parietal). For EVC, there was 1 node located in the right temporal lobe and 13 nodes located in the left hemisphere (2 were frontal, 5 temporal, 3 parietal, 2 insular, and 1 hippocampus). For CC, there were 21 nodes located in the right hemisphere (4 were frontal, 4 temporal, 2 parietal, 2 insular, 2 limbic, 1 occipital, 1 hippocampus, 4 basal ganglia, and 1 thalamus) and 2 nodes located in the left hemisphere (1 was frontal and 1 was in the thalamus). For NE, there were 44 nodes located in the right hemisphere (7 were frontal, 14 temporal, 4 parietal, 2 insular, 2 limbic, 1 occipital, 2 amygdala, 2 hippocampus, 5 basal ganglia, and 5 thalamus) and 1 node located in the left parietal lobe

Comparison of the BC, EVC, CC, and NE of the complete adjacency matrices in the theta band of the LPs and RPs was able to identify nodes, which are shown in Figure 5, that were significantly different (*p* < .05, FDR‐adjusted) between the two patient groups. The CC and NE identified nodes primarily in the right hemisphere while the BC and EVC identified nodes primarily in the left hemisphere. For BC, there were 8 nodes located in the left hemisphere (6 were temporal). For EVC, there was 1 node located in the right temporal lobe and 13 nodes located in the left hemisphere (5 were temporal and 1 was in the hippocampus). For CC, there were 21 nodes located in the right hemisphere (4 were temporal and 6 subcortical) and 2 nodes located in the left hemisphere (1 was frontal and 1 was in the thalamus). For NE, there were 44 nodes located in the right hemisphere (14 were temporal and 14 subcortical) and 1 node located in the left parietal lobe.

The NBS network had nodes in 20 brain areas (16 in the right hemisphere and 4 in the left hemisphere) while BC identified significant nodes in six brain areas (all left), EVC in nine brain areas (1 right and 8 left), CC in 17 brain areas (15 right and 2 left), and NE in 18 brain areas (17 right and 1 left). The number of brain areas that matched was 13 between the NBS network and the CC (12 right and 1 left), 13 between the NBS network and the NE (all right), 14 between the CC and NE (all right), 1 between the CC and EVC (all right), 1 between the NE and EVC (all right), and 4 between the BC and EVC (all left). The number of nodes in each of these brain areas that were in the NBS network and that were significant for the local graph measures is shown in Table 4.

**Table 4:** The number of significant nodes (p < .05, false discovery rate [FDR]‐adjusted) in each brain area identified via comparison of the betweenness centrality (BC), eigenvector centrality (EVC), clustering coefficient (CC), and nodal efficiency (NE) of the complete adjacency matrices in the theta band of patients with right‐hemispheric focal epilepsy (RPs) and patients with left‐hemispheric focal epilepsy (LPs) along with the number of nodes in each brain area of the subnetwork identified using network‐based statistics (NBS) with a 3.45 primary threshold, 5,000 random permutations, and a p < .05 significance level (family‐wise error rate [FWER]‐corrected) on the connection strengths of the complete, un‐thresholded adjacency matrices of RPs and LPs in the theta band

Brain area (right)	NBS	Local graph measures			
		CC	NE	BC	EVC
*Right middle frontal gyrus*	*1*	*1*	*1*	0	0
*Right orbital gyrus*	*1*	*1*	*3*	0	0
Right precentral gyrus	1	0	0	0	0
*Right inferior frontal gyrus*	0	*2*	*3*	0	0
*Right superior temporal gyrus*	*1*	*1*	*3*	0	0
*Right middle temporal gyrus*	*2*	*1*	*3*	0	0
*Right inferior temporal gyrus*	*6*	*1*	*3*	0	0
*Right fusiform gyrus*	*2*	0	*1*	0	0
*Right parahippocampal gyrus*	0	*1*	*4*	0	*1*
*Right superior parietal lobe*	*1*	0	*1*	0	0
*Right inferior parietal lobe*	*2*	*1*	0	0	0
*Right precuneus*	*1*	*1*	*3*	0	0
*Right insular gyrus*	*3*	*2*	*2*	0	0
*Right cingulate gyrus*	*1*	*2*	*2*	0	0
*Right medioventral occipital cortex*	*2*	*1*	*1*	0	0
Right lateral occipital cortex	1	0	0	0	0
Right amygdala	0	0	2	0	0
*Right hippocampus*	0	*1*	*2*	0	0
*Right basal ganglia*	*1*	*4*	*5*	0	0
*Right thalamus*	*2*	*1*	*5*	0	0

Brain area (left)	NBS	Local graph measures			
		CC	NE	BC	EVC
Left superior frontal gyrus	1	0	0	0	0
*Left orbital gyrus*	*1*	*1*	0	0	0
**Left inferior frontal gyrus**	0	0	0	**1**	**1**
Left middle frontal gyrus	0	0	0	0	1
**Left superior temporal gyrus**	0	0	0	**3**	**4**
**Left middle temporal gyrus**	0	0	0	**1**	**1**
Left inferior temporal gyrus	0	0	0	1	0
Left fusiform gyrus	0	0	0	1	0
**Left postcentral gyrus**	0	0	0	**1**	**2**
Left inferior parietal lobe	0	0	0	0	1
Left precuneus	0	0	1	0	0
Left insular gyrus	0	0	0	0	2
Left medioventral occipital cortex	1	0	0	0	0
Left lateral occipital cortex	2	0	0	0	0
Left hippocampus	0	0	0	0	1
Left thalamus	0	1	0	0	0

*Note:* The NBS network had nodes in 20 brain areas (16 in the right hemisphere and 4 in the left hemisphere) while BC identified significant nodes in 6 brain areas (all left), EVC in 9 brain areas (1 right and 8 left), CC in 17 brain areas (15 right and 2 left), and NE in 18 brain areas (17 right and 1 left). The number of brain areas that matched was 13 between the NBS network and the CC (12 right and 1 left), 13 between the NBS network and the NE (all right), 14 between the CC and NE (all right), 1 between the CC and EVC (all right), 1 between the NE and EVC (all right), and 4 between the BC and EVC (all left). *Brain areas that matched between any of the NBS network, CC, NE, and EVC are in italics while brain areas that matched between the BC and EVC are in boldface.*

Abbreviations: CC, clustering coefficient; BC, betweenness centrality; EVC, eigenvector centrality; NBS, network‐based statistics; NE, nodal efficiency.

## 4 DISCUSSION

Three global graph measures (i.e., global efficiency [GE], characteristic path length [CPL], and transitivity [T]) were used in this study to compare the intra‐hemispheric brain networks of HCs, patients with left‐hemispheric focal epilepsy (LPs), and patients with right‐hemispheric focal epilepsy (RPs). This was accomplished by using beamformer source reconstruction with resting‐state MEG (rs‐MEG) data to derive virtual electrode (VE) time‐series for the region‐of‐interest (ROI) centroids of the Brainnetome atlas, after which the debiased weighted phase lag index (dwPLI) was used as a measure of functional connectivity (FC) between the ROI centroids. Only the global graph measures of the right intra‐hemispheric network were significantly different between RPs and LPs; all three in the theta band, and the GE and T in the delta band. The global graph measures of the right intra‐hemispheric network in the theta band were also significantly different between HCs and RPs but not between HCs and LPs. Based on these results, the complete brain networks (i.e., composed of both the intra‐ and interhemispheric networks) in the theta band of the patients were analyzed with network‐based statistics (NBS) to identify other subnetworks that may be different between LPs and RPs. The brain networks in the theta band were also analyzed with four local graph measures (i.e., nodal efficiency [NE], clustering coefficiency [CC], betweenness centrality [BC], and eigenvector centrality [EVC]) to identify brain regions that may have different network properties for LPs and RPs.

The NBS subnetwork was primarily composed of intra‐hemispheric connections within the right hemisphere, which is consistent with the global graph measures in the theta band being significantly different between LPs and RPs for the right intra‐hemispheric network and not for the left intra‐hemispheric network. Additionally, the graph measures that are local variants of the global graph measures (i.e., NE for GE and CC for T) were primarily significant for nodes located in the right hemisphere, which also agrees with the results obtained with the global graph measures and NBS. However, the local graph measures that indicate centrality (i.e., BC and EVC) were primarily significant for nodes located in the left hemisphere. For the NBS subnetwork and the local graph measures, the nodes were primarily located in the temporal lobe. This result may be due to all the patients having either TLE or TELE. Other studies have also shown differences between the functional networks of RPs and LPs in the theta band on a local level. An EEG study found that, for classification of patients with left or right TLE, the directed connection strengths in the theta band from the right to the left hippocampus and the right medial temporal pole (MTP) to the right amygdala were important features (Verhoeven et al., 2018). A MEG study found that the connection strength in the theta band between the right hippocampus and left middle frontal lobe (MFL) was significantly greater for patients with right mesial TLE than for patients with left mesial TLE and epilepsy patients with focal cortical dysplasia (FCD) (Jin & Chung, 2015).

The GE and T of the right intra‐hemispheric network in the theta band were significantly greater for RPs than both LPs and HCs while the CPL was significantly lower, which indicates a greater functional integration and segregation in the right (affected) intra‐hemispheric brain networks of RPs in the theta band. The increased segregation is comparable with a study using both EEG and MEG that reports a greater mean CC in the theta band for the functional networks of patients with focal epilepsy than those of HCs (Horstmann et al., 2010). Although the increased integration is not comparable with Horstmann et al., which reports a greater theta CPL for the functional networks of patients with focal epilepsy (Horstmann et al., 2010), it is comparable with a MEG study that reports a lower mean eccentricity in the theta band for the functional networks of patients with frontal lobe epilepsy (Niso et al., 2015). Both the increased integration and segregation may also be comparable with a MEG study that reports a greater GE, mean CC, and mean nodal strength in the 5–14 Hz frequency range for the functional networks of epilepsy patients with absence seizures (Chavez et al., 2010). However, these studies neither distinguishes between RPs and LPs nor between unaffected and affected intra‐hemispheric networks. Other studies have also shown altered global characteristics for the functional networks of patients with epilepsy in the theta band. An EEG study found that patients with epilepsy had a greater mean connection strength in the theta band than HCs (Douw, de Groot, et al., 2010) while a MEG study found that patients with tumor‐related epilepsy had a positive correlation between seizure frequency and both mean connection strength (significant) and CPL (nonsignificant) in the theta band (Douw et al., 2010).

The altered functional networks of patients with epilepsy in the theta band may be related to the origin and regulation of theta oscillations (Douw, van Dellen, et al., 2010). Although theta oscillations have been thought to originate from the hippocampus and then spread to the outer cortical layers, studies have reported that other brain regions may generate theta oscillations during certain cognitive states (Mizuki, Tanaka, Isozaki, Nishijima, & Inanaga, 1980). Gamma‐aminobutyric acid (GABA)‐ergic interneurons in the hippocampus may be responsible for the regulation of theta oscillations (Klausberger et al., 2003; Klausberger & Somogyi, 2008). Animal studies have reported that induction of generalized epilepsy in rats via blockade of GABA receptors altered patterns of theta oscillations (Mackenzie, Medvedev, Hiscock, Pope, & Willoughby, 2002), that perturbation of hubs formed by GABA‐ergic interneurons altered network synchronization in the hippocampus of rats and mice (Bonifazi et al., 2009), and that induction of TLE in rats disrupted hippocampal theta resonance and phase lead (Marcelin et al., 2009). A study found that generation of hippocampal theta oscillations due to medial septal neurons produced an anti‐epileptic effect in rats with TLE (Colom, Garcia‐Hernandez, Castaneda, Perez‐Cordova, & Garrido‐Sanabria, 2006). As can be seen in a recent review article (Kitchigina, 2018), other studies have shown that TLE and Alzheimer's disease (AD) result in alterations of theta and gamma oscillations in networks involving the hippocampus and other brain regions. Verhoeven et al. found that the directed connection strength in the theta band from the right to the left hippocampus was an important feature for classification of patients with right or left TLE and that the directed connection strengths in the theta band from the right to the left hippocampus and from the right hippocampus to the right amygdala were important features for classification of HCs and patients with TLE (Verhoeven et al., 2018). In our study, the CC and NE in the theta band identified nodes that were significantly different between RPs and LPs in the right hippocampus while the EVC in the theta band identified a significant node in the left hippocampus.

The GE of the right intra‐hemispheric network in the delta band was significantly lower for LPs than both RPs and HCs while the T was significantly greater for RPs than both LPs and HCs, which indicates a lower functional integration in the left (unaffected) intra‐hemispheric brain networks of LPs and a greater functional segregation in the right (affected) intra‐hemispheric brain networks of RPs in the delta band. The increased segregation and decreased integration is comparable with Horstmann et al., which reports a greater CPL and mean CC in the delta band for the functional networks of patients with focal epilepsy than those of HCs (Horstmann et al., 2010). However, Horstmann et al. neither distinguishes between RPs and LPs nor between unaffected and affected intra‐hemispheric networks.

All three global graph measures of both intra‐hemispheric networks were significantly different between the HCs and both patient groups in the low beta, high beta, and low gamma bands; the right and left CPL and the right T in all three bands, and the right and left GE and the left T in the beta bands. The GE and T were greater while the CPL was lower for the patients than the HCs, which indicates a greater functional integration and segregation in the brain networks of patients with focal epilepsy in the higher frequency bands. The increased integration is comparable with results obtained using MEG FC analysis to compare HCs and patients with generalized epilepsy in the beta and gamma bands (Niso et al., 2015) and using fMRI FC analysis to compare HCs and patients with TLE (Doucet et al., 2015; Garcia‐Ramos et al., 2016). However, the increased segregation is not comparable with the results obtained using fMRI FC analysis, which reports that the patients had lower segregation than the HCs (Doucet et al., 2015; Garcia‐Ramos et al., 2016). Although generalized and focal epilepsy are two different types of epilepsy, both are disorders of abnormal brain networks (Engel Jr. et al., 2013). Therefore, their network topologies may share some characteristics. Our study as well as the previously mentioned studies suggest that one of these characteristics may be the functional integration in the higher frequency bands.

Most of the aforementioned studies on patients with epilepsy examined the global characteristics of the network of the entire brain. Douw et al. found that the combined left and right intra‐hemispheric networks along with the left temporo‐occipital, right temporo‐occipital, right fronto‐temporal, and left temporal networks of patients with tumor‐related epilepsy had a significant positive correlation between seizure frequency and mean connection strength in the theta band (Douw, van Dellen, et al., 2010). However, their sample was composed of patients with generalized epilepsy as well as patients with focal epilepsy, and they did not compare RPs and LPs. Another study using EEG FC analysis found that, for the CPL and mean CC in the alpha band, the difference between the left and right intra‐hemispheric fronto‐temporal networks was significantly more positive for patients with left fronto‐temporal epilepsy and significantly more negative for patients with right fronto‐temporal epilepsy than for HCs with the affected networks of the patients having higher values than the unaffected (Vecchio et al., 2015). This is not comparable with our study, which found a significant difference between the right intra‐hemispheric graph measures of the RPs and LPs in the theta and delta bands rather than the alpha and did not find a significant difference between the left intra‐hemispheric graph measures of the RPs and LPs.

When used as input features of a Naïve‐Bayes classifier, the global graph measures of the right intra‐hemispheric network in the theta band were able to classify the LPs and RPs with a 78.0% accuracy and an AUC of 0.869 for both patient groups while the GE in the theta, low beta, and high beta bands and the CPL and T in the theta, low beta, high beta, and low gamma bands of the right intra‐hemispheric network were able to classify the three groups with a 75.5% accuracy and an AUC of 0.988 for the HCs, 0.837 for the LPs, and 0.854 for the RPs. The performance of these classifiers indicates that the global graph measures of the right intra‐hemispheric network may be useful not only for the diagnosis of focal epilepsy but also for lateralization. This may be important for patients with focal epilepsy who are drug‐resistant and MRI normal, particularly if there is a lack of or an ambiguity in the seizures or IEDs recorded during MEG, EEG, or other neuroimaging modalities. Studies report that only about 30–50% of patients with epilepsy have IEDs on their first EEG recording (Douw, de Groot, et al., 2010; King et al., 1998) and that about 2–18% of patients never have IEDs during their EEG recordings (Douw, de Groot, et al., 2010; Marsan & Zivin, 1970; Noachtar & Remi, 2009). For MEG, the average sensitivity in detecting clinically relevant IEDs is ~75% (Carrette & Stefan, 2019; Pataraia et al., 2004; Paulini et al., 2007; Stefan et al., 2003). The application of machine learning algorithms to graph analysis of resting‐state networks for assistance in classification of patients with epilepsy is a rapidly growing field, as can be seen in many studies (Babajani‐Feremi, Noorizadeh, Mudigoudar, & Wheless, 2018; Douw, de Groot, et al., 2010; Nissen et al., 2017; Nissen et al., 2018; Verhoeven et al., 2018).

## 5 LIMITATIONS

There are some limitations to this study. In particular, because most of the patients were medicated with anti‐epileptic drugs (AEDs), the influence of the AEDs on their brain networks is a potential confound. This may be especially true for comparisons between the HCs and the patients. Several studies have shown changes in functional networks that are associated with AED use (Haneef, Levin, & Chiang, 2015; van Veenendaal et al., 2017). However, it is standard clinical practice for our center to gradually wean patients off their AEDs before the time of MEG data collection. Additionally, the patient groups were matched (*p* > .05) for the number of different AED types that they were medicated with (see Table 1) and the distribution of the different AED types across the two groups was relatively uniform. Therefore, the variability in the FC due to the AEDs was minimized. Another limitation of this study was that, due to a lack of availability, the HCs were from a slightly older age group than the patients. To account for this heterogeneity, analysis of covariance (ANCOVA) was performed to test whether the effect of age on the global graph measures was significant. The effect of age was shown to not be significant (*p* > .05, FDR‐adjusted) in the delta, theta, alpha, low beta, and high beta bands.

An additional limitation was that resting‐state MEG data were collected during an eyes‐open (EO) condition for the HCs and eyes‐closed (EC) condition for the patients. This is because the MEG data were collected under two different protocols: a clinical protocol for the patients and a research protocol for the controls. Resting‐state MEG data under research protocol are collected during an EO condition. However, resting‐state MEG data under clinical protocol are collected during an EC condition in our center, which is important for patients with epilepsy who are often developmentally delayed and have difficulty following instructions to minimize artifacts due to eye movements and blinking. Tan et al. compared the functional networks of HCs during EO and EC resting‐state conditions (Tan, Kong, Yang, Jin, & Li, 2013). They found that, in the theta band, the mean CC of the entire brain network decreased only by about 13% from an EC to an EO condition. In our study, as shown in Figure 2 and Figure 3, the transitivity (a variant of the mean CC) of the right intra‐hemispheric network in the theta band was significantly less by more than 70% (*p* < .005, FDR‐adjusted) for the controls (under EO) than for the patients with right‐hemispheric focal epilepsy (under EC). Therefore, because the 70% difference observed in our study is much more than the 13% difference reported by Tan et al., it is unlikely that our results in the theta band would be greatly impacted by the different EC and EO conditions.

## 6 CONCLUSIONS

To investigate functional and effective networks of patients with epilepsy, global and local graph measures have been used to extract useful features from the network of the entire brain. Because focal epilepsy originates within networks limited to one cerebral hemisphere, using global graph measures to characterize intra‐hemispheric networks rather than the network of the entire brain may be able to reveal additional information about the brain networks of patients with focal epilepsy. In this study, global graph measures based on FC analysis with rs‐MEG data were able to distinguish between the intra‐hemispheric brain networks of HCs, LPs, and RPs, particularly in the theta band. The GE, CPL, and T of the right intra‐hemispheric network in the theta band were significantly different between the RPs and both the LPs and HCs while the GE, CPL, and T of both intra‐hemispheric networks in the higher frequency bands were significantly different between the HCs and both the LPs and RPs. When used as input features of a Naïve‐Bayes classifier, the global graph measures of the right intra‐hemispheric network were able to classify the LPs and RPs as well as all three groups. In the theta band, NBS was able to identify a subnetwork that differs between LPs and RPs while local graph measures were able to identify brain regions that have different network properties for LPs and RPs. The NBS subnetwork was primarily composed of intra‐hemispheric connections within the right hemisphere while the CC and NE identified significant nodes primarily within the right hemisphere. However, the BC and EVC identified significant nodes primarily within the left hemisphere. Overall, the results of this study indicate that the global graph measures of the MEG‐based right intra‐hemispheric network may be useful for the diagnosis and lateralization of focal epilepsy.

## CONFLICT OF INTEREST

The authors declare no conflict of interest.

## Data Availability

The anonymized data and code are available from the corresponding author upon reasonable request, consistent with the policy of and subject to confirmation by the University of Tennessee Health Science Center Ethics Committee.
